# Novel Therapeutic Approaches for Treatment of Diabetic Retinopathy and Age-Related Macular Degeneration

**DOI:** 10.3390/vision9020035

**Published:** 2025-04-17

**Authors:** Deokho Lee, Soo Jin Kim, Junyeop Lee

**Affiliations:** Department of Ophthalmology, Asan Medical Center, University of Ulsan College of Medicine, Seoul 05505, Republic of Korea

**Keywords:** age-related macular degeneration, diabetic retinopathy, ischemic retinopathy, vitamins, hypoxia-inducible factors, cell therapy, lipid metabolism

## Abstract

Retina, a light-sensitive layer of tissue of the eye, requires high levels of oxygen for its physiology. Retinal ischemia occurs due to inadequate supply of blood to the retina and choroid. Retinal ischemia is implicated in the development or progression of many ocular diseases, such as diabetic retinopathy (DR) and age-related macular degeneration (AMD). To date, anti-vascular endothelial growth factor (VEGF) treatment has been widely used to manage neovascular diseases associated with retinal ischemia. Nonetheless, a substantial number of patients with DR or AMD still suffer from incomplete response and adverse effects related to its therapy with limitations. Therefore, research scientists have been developing and finding novel treatments to protect against or prevent vision loss in those diseases. In this review article, we summarize the recent novel therapeutic approaches for the treatment of ischemic retinopathy (e.g., cell therapy, advanced molecular targeting, or drug delivery). This summary enables further research to obtain more solid evidence of novel effective drug development in retinal ischemic diseases.

## 1. Introduction

The retina is one of the most oxygen-consuming tissues in our body. The retina is vulnerable to oxygen insufficiency. Retinal hypoxia can physiologically and pathologically affect retinal metabolic status [1]. Retinal ischemia is a pathologic condition that occurs due to a lack of necessary supply of blood to the retina [2]. Development or progression of ocular diseases, such as diabetic retinopathy (DR), age-related macular degeneration (AMD), and glaucoma, is highly related to retinal ischemia [2]. Chronic and unresolved retinal ischemia ultimately results in irreversible vision loss [2]. To understand the disease development and progression, experimental models that mimic the pathologic condition of the disease are needed [3]. Furthermore, developing or finding novel treatments is highly important under experimental conditions.

Many experimental models have been used to understand pathologic conditions in retinal diseases. For instance, streptozotocin (STZ), a compound selectively toxic to pancreatic islets, is injected into rodents to cause diabetic conditions for studying DR [4,5]. The db/db mouse is a genetic experimental model for spontaneous type 2 diabetes, and several DR phenotypes (such as vascular damage, inflammation, and retinal neuronal degeneration; *described below in detail*) could be seen in this model [6,7]. Akita and Akimba mice are also genetic experimental models for type 1 diabetes and DR [8,9,10].

Laser burns are induced in rodent eyes to disrupt Bruch’s membrane, leading to abnormal blood vessel growth from the choroid into the subretinal area, termed choroidal neovascularization (CNV) [11], detected in wet AMD. Very low-density lipoprotein receptor knockout mice (*Vldlr*^−/−^) are reported to experience subretinal neovascularization [12], resembling features of retinal angiomatous proliferation (a subtype of wet AMD). Not only in vivo models but also in vitro systems (e.g., retinal endothelial cells or pericytes, ARPE-19, or 661W cells; 3D organoids [13,14,15,16,17,18]) foster experimental ophthalmology research and further drug development in this field.

To date, effective treatment options for neovascular retinal disease associated with retinal diseases are suggested but limited. Anti-vascular endothelial growth factor (VEGF) treatment has been widely used in the fields of ophthalmology and oncology [19,20,21]. VEGF is a strong angiogenic factor for vascular endothelial cells [22,23]. VEGF is produced by many types of cells (including abnormal cells, such as tumors) [22,23]. Many researchers have found that anti-VEGF treatment can inhibit neovascularization in the eye [19,21]. Furthermore, visual acuity can be improved by anti-VEGF treatment [24]. However, despite the principal blockade of VEGF, anti-VEGF treatment can fail from the beginning or following an initial successful treatment period [25,26]. Reduced response to anti-VEGF treatment may also include the development of fibrosis [27,28], potential resistance to the therapy itself, or involvement of other pro-angiogenic factors for neovascularization.

Other strategies to slow disease progression or protect against vision loss include laser therapy, steroid treatment, and surgery [29]. While effective in preventing vision loss, each strategy has side effects including potential complications [30,31,32,33]. Furthermore, these invasive strategies focus on the advanced stages of the disease. Therefore, novel therapeutic approaches to overcome these limitations are considered.

In this review article, we aim to summarize the current pathophysiology of retinal ischemia in DR and AMD. We further present interesting therapeutic approaches for retinal diseases (Table 1). Recent potentials for cell therapy, advanced molecular targeting, and drug delivery are discussed as effective treatment candidates in ischemic retinopathy.

## 2. Diabetic Retinopathy

### 2.1. Pathophysiology

DR is one of the major complications of diabetes affecting the eyes. The high level of blood glucose (termed hyperglycemia) in diabetes affects the retinal microvascular system even from the early disease stage [34]. Hyperglycemic vascular damage occurs due to various pathologic biochemical pathways, such as the accumulation of advanced glycation end products (AGEs), induction of protein kinase C (PKC), and the polyol and hexosamine pathways [29].

At the early stage of DR, pericyte loss is considered one of the pathologic hallmarks [35,36]. Pericyte physiologically provides vascular stability and controls endothelial proliferation, which implies that interactions between pericyte and endothelial cells are highly important for vascular homeostasis [37]. Several key mediators/factors for their interactions have been suggested: transforming growth factor-β (TGF-β), angiopoietin 1/angiopoietin 2 (Ang1 and Ang2) and Tie-2, platelet-derived growth factor receptor-β-(PDGFR-β) and PDGF, and VEGF [38,39,40]. In fact, pericyte dropout accelerates, rather than directly causes, DR progression by destabilizing retinal vascular endothelial cells [36]. Preserving pericyte attachment or enhancing endothelial cell-pericyte interactions may help mitigate DR progression by maintaining the blood-retinal barrier integrity. Although more studies are needed, modulations of those factors as well as cell-to-cell interactions could be considered to effectively manage DR progression at the early stage.

Pericyte dropout in diabetes may have consequences on capillary remodeling, leading to retinal ischemia. As retinal neovascularization occurs at this severe stage of DR [41], many therapeutic intervention studies have focused on understanding the development of retinal neovascularization and finding critical therapeutic molecular targets to intervene in its development. Under this condition, hypoxia-inducible factors (HIFs) are considered strong transcription factors to activate various pro-angiogenic and pro-inflammatory factors (including VEGF), which leads to retinal neovascularization and further vascular damage [1,42].

Along with vascular damage, retinal inflammation and neurodegeneration are detected in DR. Many chemokines and cytokines are suggested to have detrimental roles in the pathophysiology of DR: monocyte chemoattractant protein-1 (MCP-1/CCL2), tumor necrosis factor-alpha (TNF-α), and the interleukin (IL) family [43,44,45]. Meanwhile, chronic hyperglycemia causes mitochondrial oxidative stress in retinal neuronal and non-neuronal cells, leading to retinal metabolic dysregulation [46,47,48]. Entire retinal layers are generally affected by chronic hyperglycemia [49,50]. These vicious cycles (neovascularization, inflammation, oxidative stress, and neurodegeneration) eventually cause irreversible vision loss.

Other metabolic factors might also affect the progression of DR. In clinical studies, fenofibrate, a lipid-lowering/modulating drug, reduces the risk of progression of DR [51,52]. Although a relationship between lipid levels and the progression of DR has not been clearly determined yet, abnormal serum levels of lipids (such as low-density lipoproteins, high-density lipoproteins, total cholesterol, triglycerides, and the apolipoprotein family) might be the risk factors for the progression of DR [53,54,55]. Regarding this metabolic aspect, various preclinical studies have been actively conducted.

### 2.2. Therapeutic Approaches

Many researchers have aimed to manage DR development and progression through distinct therapeutic strategies. This section aims to provide a comprehensive review of the next therapeutic approaches, mainly focusing on retinal metabolism and regeneration in DR.

#### 2.2.1. Peroxisome Proliferator-Activator Receptor Alpha

Peroxisome proliferator-activator receptor alpha (PPARα) is one of the nuclear receptor family of PPARs. PPARα is a key regulator of lipid oxidation, and its expression is detected in many tissues, including the retina [56]. Fenofibrate is a well-known PPARα agonist. Hu et al. found that STZ-induced diabetic rats, Akita mice, and db/db mice have a decrease in retinal PPARα expression [57]. Furthermore, diabetic mice with PPARα knockout show more severe retinal vessel impairment and higher vascular leakage compared with wild-type diabetic mice. Although cell types important for therapeutic PPARα activation have not been clearly determined, retinal PPARα seems important to modulate the progression of DR.

Ding et al. similarly found that fenofibrate treatment ameliorates retinal acellular capillary formation and pericyte loss in STZ mice [58]. Furthermore, diabetic PPARα knockout mice show more severe retinal capillary and pericyte damage, compared with diabetic wild-type mice.

Yuan et al. focused on the roles of microglial PPARα in DR [59]. Microglia-specific PPARα deletion exacerbates retinal pericyte loss in diabetes. Furthermore, its deficiency leads to microglial pro-inflammatory polarization under diabetic conditions. This provides therapeutic insight into microglial PPARα activation against DR progression.

Dong et al. found reductions in PPARα levels in diabetic monocytes from patients with diabetes [60]. A PPARα-dependent effect of fenofibrate on the inhibition of monocyte activation in diabetes was also detected. This further provides important roles of PPARα in monocytes during DR progression.

Protective and preventive effects of fenofibrate and fenofibric acid have been gradually reported under various experimental conditions, and well-discussed in previous reviews [61,62]. As a next-generation therapeutic approach, Huang et al. increased the drug availability to the retina by developing a novel nano-emulsion fenofibrate eye drop [63]. This eye drop is well-delivered to the retina/vitreous and exerts therapeutic effects in STZ-induced rats. Furthermore, the eye drop is not toxic to the eye. Although more studies are needed, nanotechnology could be well-applied for DR treatment.

Hanaguri et al. also examined the therapeutic effects of this fenofibrate nano eye drop in diabetic mice [64]. Their data suggested that the fenofibrate nano eye drop prevents retinal glial dysfunction through the phosphorylation of PPARα with the improvement of retinal blood flow dysregulation in DR. Its fenofibrate ophthalmic formulations are proven to be well-delivered to the retina. As the use of nanoparticles needs further investigations regarding its suitable size, surface charge, solubility, and degradation capability, more experimental conditions’ outcomes should be stacked. Taken together, as fenofibrate’s therapeutic effects on the eye/retina are not limited to systemic activation of PPARα to reduce circulating lipid levels [65], the use of fenofibrate and its eye drop formulation could be applied to effectively manage DR progression with this advanced non-invasive method.

Pemafibrate is a selective peroxisome proliferator-activated receptor alpha modulator (SPPARMα). With emerging evidence, pemafibrate is superior to fenofibrate in terms of drug efficacy and safety for patients with dyslipidemia [66,67,68]. In preclinical studies for ophthalmology, pemafibrate treatment shows neuroprotective, anti-inflammatory, antioxidative stress, and anti-neovascularization effects in rodent models of DR and other ischemic retinopathies, well-discussed in the previous literature [69].

Although this research is not directly related to retinopathy, Murakami et al. found that pemafibrate and sodium-glucose cotransporter 2 (SGLT2) inhibitor combination treatment improves pathological progression in an experimental rodent model of non-alcoholic steatohepatitis [70]. Similarly positive effects of the combination treatment have been examined [71]. SGLT2 modulation has received significant attention for treating systemic metabolic diseases [72,73]. Therefore, the PPARα activation and SGLT2 inhibition combination strategy could be used for treating various forms of ischemic retinopathy as retinopathy is highly related to systemic metabolic diseases [74,75,76].

#### 2.2.2. Vitamins

Vitamins (from vitamin A to vitamin D) are organic compounds essential for maintaining metabolic function. Although vitamins are not generally defined as drugs (but supplements), vitamins have been recently recommended to manage various metabolic diseases and disorders [77,78]. Most studies on the use of vitamins in DR are to find the association between systemic vitamin levels and the development or progression of DR.

Choi et al. found that high levels of vitamin A are associated with a low risk of DR in a population-based epidemiological study from the Korean national health and nutrition examination survey [79]. Rostamkhani et al. also demonstrated that vitamin A levels are related to the severity of retinopathy in diabetes [80].

Cinici et al. found that lower blood thiamine pyrophosphate concentrations are associated with a higher risk of DR [81]. Thiamin (vitamin B1) and its derivatives might be targeted for DR management.

Horikawa et al. demonstrated that high vitamin B6 intake is associated with a lower incidence of DR in Japanese people with type 2 diabetes, examined in a nationwide cohort (the Japan Diabetes Complications Study; JDCS) [82]. Yin et al. explored the association between vitamin B6 turnover rate and DR in a cross-sectional study and found a positive relationship between high levels of vitamin B6 turnover and an increased risk of DR [83]. Ruan et al. showed vitamin B6 intake is negatively associated with the risk of DR, and in patients with DR, a higher intake of vitamin B6 is associated with a lower risk of all-cause death and cardiovascular disease-related death [84]. Other vitamins (e.g., vitamins C, E, and D) might have therapeutic effects on DR management based on the previous literature [85,86,87,88].

In experimental studies, Reddy et al. found that vitamin B12 supplementations histologically reduce retinal damage in diabetic rats through endoplasmic reticulum stress-mediated retinal cell death [89]. Although it is not directly related to diabetes-induced retinal damage, Wang et al. found that vitamin B6 supplementations also histologically protect retinal neurons against ischemic injury in primates [90]. As vitamin B is known as a scavenger of reactive oxygen species (ROS) [91], its supplementations may work on retinal protection as antioxidants. Although vitamin supplementations are generally essential for systemic physiology and development, the direct therapeutic effects of dietary intake of each vitamin in ocular diseases (including DR) should be well understood along with accumulating experimental evidence under disease conditions.

#### 2.2.3. Cell Therapy

Cell-based therapy is one of the novel strategies to manage DR. As chronic hyperglycemia in diabetes affects the entire layers in the retina, depending on the therapeutic purpose and the timepoint of DR, the target cells could be different. Using stem/progenitor cells is considered an attractive approach, in that targeted differentiation of cells is inducible into specialized cell types [92,93].

Rong et al. suggested that intravenous transplantation of human embryonic stem cell-mesenchymal stromal cells ameliorates retinal and microvascular damage in diabetic mice [94]. Cheung et al. found that intravitreal injection of human CD34^+^ bone marrow stem cells exerts protective effects on the superficial retinal capillary plexus layers in STZ mice [95]. Scalinci et al. suggested a potential effect of intravitreal human placental stem cell implants in inhibiting DR progression through changes in neuroprotective growth factors in the vitreous [96].

Although therapeutic evidence for cell-based therapy is growing, its approach faces critical limitations: proper target cell differentiation, long-term functional survival of the target cells, and the potential of target cells to become unexpected tumors or evoke immune responses to affect the disease condition. Although the examination of cell-based therapy is not directly focused on DR research, large experimental in vivo models (e.g., cats, dogs, and pigs) have been used for stem cell therapy to cure retinitis pigmentosa and photoreceptor loss [97]. Therefore, fundamental animal-based preclinical studies for future human trials should be performed, as the feasibility of preclinical data for clinical applications is not directly guaranteed, which is a huge challenging barrier.

#### 2.2.4. Hypoxia-Inducible Factors

HIFs are composed of α and β subunits, and three isoforms have been discovered (HIF-1, HIF-2, and HIF-3). HIFs are mainly involved in cellular oxygen homeostasis [98]. While HIF-α is rapidly degraded under normoxia, its expression could be well-detected under hypoxia, followed by its translocation into the nucleus to increase various HIF-target gene expressions [1]. The roles of the upregulated target genes are related to angiogenesis, inflammation, glucose metabolism, and cell death and survival.

Retinal HIFs are activated during DR progression and lead to retinal neovascularization. Wert et al. found that vitreous samples from DR patients contain elevated HIF-1α levels [99]. Lim et al. found that HIF-1α is detected more often and more intensely in diabetic preretinal membranes, in comparison with that in nondiabetic idiopathic epiretinal membranes [100]. Mazzeo et al. found that HIF-1α expression increased in extracellular vesicles from patients with DR [101]. Therefore, many researchers have focused on finding or developing strong HIF inhibitors and examining their efficacies to potentially inhibit pathologic vessel growth in the retina.

To study retinal neovascularization in DR, suitable experimental diabetic models that reflect the human DR condition have been highly required. Unfortunately, easily accessible, inducible, and cost-effective rodent DR models that generate diabetes-induced proliferative retinopathy phenotypes with a certain level of model stability do not exist, while genetically (e.g., VEGF and insulin-like growth factor 1; IGF-1) modified mice could be considered for the substitution, in that retinal neovascularization might occur [102,103]. As pericyte loss induces vascular dysfunction to finally trigger ischemic conditions in the diabetic retina, STZ mice should have retinal neovascularization at some chronic time points. However, STZ mice are generally known to have a lack of hypoxic/ischemic phenotypes in the retina, although pericyte loss is reported [104]. This discrepancy should be further studied.

Under this limited circumstance, oxygen-induced retinopathy (OIR), primarily developed and used as a rodent model for studying retinopathy of prematurity [105], is widely and indirectly used to study proliferative DR because of pathologic retinal angiogenesis during the model development. In this OIR model, HIFs (HIF-1 and HIF-2) are highly upregulated at the hypoxic stage, and HIF inhibition consistently shows reductions in retinal neovascularization [106,107,108].

Many HIF inhibitors (e.g., topotecan, doxorubicin, 2-azahypoxanthine, digoxin, and acriflavine) have been confirmed to reduce retinal neovascularization in OIR models [1,109,110,111]. Zhang et al. also comprehensively demonstrated that levels of angiogenic factors regulated by HIFs remain elevated in the eyes of patients with diabetes, despite treatment with anti-VEGF therapy, and a novel HIF inhibitor (called “32-134D”) prevents retinal neovascularization in OIR mice and further reduces STZ-induced vascular hyperpermeability without retinal toxicity [112]. HIFs are selectively activated under hypoxic conditions and upregulate various angiogenic factors, including VEGF, to cause neovascularization. As targeting VEGF may not effectively block its pathologic process, inhibiting HIFs might be a better option [113]. Taken together, targeting HIFs is an effective therapeutic approach for managing DR progression.

## 3. Age-Related Macular Degeneration

### 3.1. Pathophysiology

AMD is a multifactorial disease that causes progressive vision loss. Aging, environmental cues (e.g., smoking, diet, and physical activity), and genetic factors affect the development and progression of AMD [114]. Chronic inflammation, lipid metabolic dysregulation (including drusen), and oxidative stress are implicated in its pathogenesis [115,116].

AMD generally affects photoreceptors, retinal pigment epithelium (RPE), Bruch’s membrane, and the choroid [115,116]. From the early AMD stage, drusen deposition and RPE dysfunction may be detected. Loss of photoreceptors and RPE begins in this stage and continues to the later stage. The later stage of AMD is characterized by geographic atrophy (GA), macular neovascularization (MNV or CNV), and fibrosis.

The RPE plays a pivotal role in retinal metabolic homeostasis by maintaining the blood-retina barrier, transporting nutrients, removing photoreceptor outer segments, and producing various factors for retinal physiology [117,118]. Therefore, RPE dysfunction or its death can lead to the accumulation of abnormal deposits, inefficiency of phagocytosis, severe inflammation, and further photoreceptor damage and loss. Other senescent cells may abnormally produce various pro-inflammatory cytokines and chemokines to affect the retina [119]. Those events can stimulate immune cells to affect retinal neurons, eventually causing pathologic consequences in AMD.

### 3.2. Therapeutic Approaches

Similar to DR treatment, many types of novel therapeutic approaches have been examined. This section aims to provide a comprehensive review of the currently tested therapeutic approaches, primarily for wet AMD cases and for some dry AMD cases.

#### 3.2.1. Antioxidants

The therapeutic effects of antioxidants have been widely examined in AMD cases. A recent study from Keenan et al. [120] showed that oral antioxidant and lutein/zeaxanthin supplementations slow GA progression to the fovea in AMD patients from the Age-Related Eye Disease Study (AREDS) and AREDS2, which implies certain vitamins and antioxidants can slow the progression of intermediate to advanced AMD. Lutein is one of the major carotenoids found in the human eye, and many natural products (e.g., egg yolks, spinach, corn, kiwi, and grapes) contain high levels of lutein [121,122]. Zeaxanthin is another common carotenoid naturally found in foods [123,124]. Arunkumar et al. found that lutein and zeaxanthin improve visual performance in *Abca4*^−/−^/*Bco2*^−/−^ mice [125]. Kamoshita et al. found that lutein suppresses RPE-choroid damage induced by extensive light exposure [126]. Lutein induces an antioxidant enzyme (e.g., superoxide dismutase SOD activity) and suppresses *Mcp-1* levels in ARPE19 cells.

Other antioxidants (such as zinc, copper, coenzyme Q10, resveratrol, and vitamins C, D, and E) also have neuroprotective effects in various experimental models of outer retinal damage [127]. Although they share similar effects in reducing oxidative stress in cells and tissues, their molecular characteristics (e.g., hydrophobicity or hydrophilicity), associated metabolic pathways, absorption capacity into our body, and necessary reaction cofactor differ. Therefore, their therapeutic uses (e.g., dose issues, administration duration and its methods, and their combination or independent effects) need more experimental evidence in various ischemic retinal pathologies for future clinical applications.

#### 3.2.2. Peroxisome Proliferator-Activator Receptor Alpha

The therapeutic roles of fenofibrate and pemafibrate have also been well-examined in AMD cases. Gong et al. found that fenofibrate treatment inhibits laser-induced CNV in mice [128]. Zhao et al. suggested fenofibrate could inhibit CNV via modulating ocular VEGF-C and VEGFR-3 expression [129]. Chen et al. showed that fenofibrate inhibits subretinal fibrosis in *Vldlr*^−/−^ mice by modulating the TGF-β signaling pathway [130]. How fenofibrate directly affects ocular VEGF and TGF signaling pathways needs more investigations using ocular cells with various ischemic stress conditions. As subretinal fibrosis is known as a cause of vision loss detected in AMD patients [131], fenofibrate could be another potential treatment for AMD patients with subretinal fibrosis.

Although the accumulating evidence is not abundant, the oral administration of pemafibrate has preventive effects on laser-induced CNV in adult mice [132]. Microglial activation is lessened, and RPE-choroid’s PPARα target genes are boosted by its treatment. At the same time, systemic fibroblast growth factor 21 (FGF21) levels are increased stably by pemafibrate treatment. Fu et al. suggested that FGF21 could have the potential to suppress CNV [133]. Accumulating evidence shows that FGF21 has neuroprotective effects or therapeutic modulatory roles in central nervous system diseases and metabolic disorders [134,135,136,137,138,139]. This implies that circulating FGF21 could be one of the important therapeutic factors to affect retinal status under pathologic conditions. This can be another intriguing therapeutic axis to be further unraveled.

The therapeutic effects of pemafibrate- and fenofibrate-mediated PPARα activation on retinal diseases have been briefly suggested based on the published literature. However, further research is needed to determine which cell types should be selectively targeted for PPARα activation, whether the therapeutic effects are systemically or locally related, what the optimal concentration is needed for the effective activation, which downstream target genes in the eye respond to pemafibrate or fenofibrate treatment, and whether there are any side effects. Along with addressing those intriguing questions, PPARα activation could be considered an interesting therapeutic approach to managing wet AMD progression.

#### 3.2.3. Cell Therapy

One of the most famous cell therapies in AMD cases might be stem cell-based autologous transplantation in AMD patients. Mandai et al. examined the feasibility of transplantation of a sheet of RPE cells derived from induced pluripotent stem cells [140]. One year after the surgery, the transplanted sheet remained intact. Along with this, a safe procedure of human embryonic stem cell-derived RPE monolayer implantation has been examined (NCT02903576). As many RPE cell therapies have been ongoing in clinical AMD studies [141], concrete conclusions are desirable in the near future.

#### 3.2.4. Hypoxia-Inducible Factors

During the development of CNV, ocular HIFs are activated and co-labelled with the abnormal vessels [142]. Therefore, HIFs have been considered strong molecular targets to inhibit CNV. Furthermore, a recent study from Sharma et al. [143] showed that VEGF inhibition could increase levels of HIF-regulated angiogenic factors by the RPE limiting the response of the wet AMD eyes to aflibercept, which implies that HIF inhibition is further necessary to overcome this inadequate response.

Babapoor-Farrokhran et al. found that HIF-1α expression colocalizes to tissue adjacent to choroidal neovascular vessels in human eyes with AMD [144]. Using a rat model of subretinal lipid peroxide-induced CNV, they further showed that its pathologic outcome could be reduced by digoxin (a well-known HIF inhibitor) treatment.

Shoda et al. found that subretinal fibrosis formation is related to HIF-1α activation (rather than HIF-2α) in RPE cells via genetic modulation studies in mice [142]. Furthermore, supplementation with taurine is suggested as useful for the prevention of and protection from AMD progression as a novel HIF inhibitor for RPE cells. Along with growing anti-aging markets, taurine has gained huge attention as a strong anti-aging supplement [145,146]. Although the mode of action of taurine in many of its beneficial health effects should be well understood, its future therapeutic use is highly considered.

Natural products-based novel HIF inhibitors (e.g., lactoferrin, rice bran, Garcinia extract, vitamin B6, and hydroxycitric acid) have also been found from various in vitro screening, and those extracts and/or compounds show anti-CNV effects, explained with HIF inhibition [147,148,149].

Iwase et al. made sustained ocular delivery of a HIF-1 antagonist to reduce CNV [150]. A HIF inhibitor is conjugated to novel copolymers of branched polyethylene glycol and poly(sebacic acid) and formulated into nanoparticles, which enables slow release of HIF inhibitor-conjugates in aqueous buffer.

Hackett et al. demonstrated that suprachoroidal delivery of acriflavine with poly(lactic-co-glycolic acid) microparticles enables long-term suppression of CNV [151]. Taken together, pharmacologic and genetic HIF inhibition and the advanced sustained drug delivery of HIF inhibitors might be applicable to manage CNV development and progression, finally preventing vision loss in AMD patients.

Although relationships of HIFs with dry AMD development are not directly discussed, Barben et al. demonstrated that increased expressions of hypoxia-related genes are detected in the aged human retina [152]. Furthermore, using photoreceptor-specific hypoxia-related gene knockout mice, they found HIF1-dependent rod photoreceptor degeneration. Other conditions show HIF- or hypoxia-dependent or independent neuroprotection in photoreceptors against oxidative stress [144,153,154,155]. This complex notion might be associated with the distinct use of experimental models for outer retinal degeneration and different observation time points. Therefore, more robust studies are highly needed regarding HIFs’ dual roles in outer retinal degeneration in dry AMD.

## 4. Conclusions

In this review article, based on preclinical evidence, novel treatment candidates (especially, antioxidants, PPARα activation, and HIF inhibition) for DR and AMD are presented and discussed.

**Figure 1:**
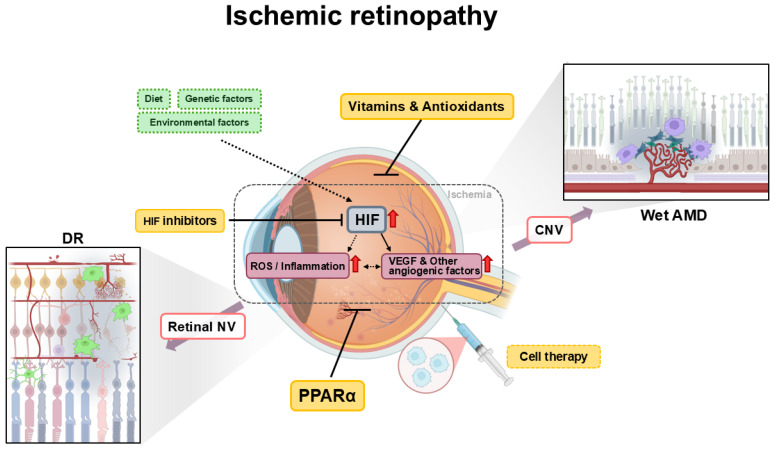
Schematic illustrations of a summary of novel therapeutics discussed in this review article for treating neovascularization (NV) and neuronal damage in diabetic retinopathy (DR) and age-related macular degeneration (AMD). DR and AMD are complex metabolic diseases. Development and progression of the diseases are affected by various risk factors (e.g., genetic factors, diet, smoking, and physical activity). Retinal ischemia is implicated in the development or progression of DR and AMD. Retinal ischemia activates hypoxia-inducible factors (HIFs) to cause NV in DR and AMD. Retinal NV is observed in proliferative DR, while macular neovascularization (MNV), including choroidal neovascularization (CNV), is detectible in wet AMD. When treated inadequately, NV eventually leads to vision loss. Pharmacologic or genetic HIF inhibition reduces NV in the retina and/or choroid. Inflammation and reactive oxygen species (ROS) also worsen DR and AMD progression. Peroxisome proliferator-activator receptor alpha (PPARα) agonists and antioxidants (including vitamins) have neuroprotective effects by reducing ocular inflammation and ROS levels. Cell therapy could be an option to reduce retinal damage or regenerate damaged cell types under disease conditions. Dotted lines: indirect associations. Straight lines: direct relationships. This image was created and edited by Soo Jin Kim via BioRender version 04, https://www.biorender.com/ (accessed on 19 March 2025). Red arrows indicate increased values.

To date, anti-VEGF therapy is considered to have limitations in clearly managing DR and AMD. Anti-VEGF therapy primarily inhibits VEGF-A receptor binding extracellularly. Some researchers have focused on tyrosine kinase inhibitors to strongly inhibit all VEGF receptor isoforms and other tyrosine kinase receptors that affect angiogenesis [156]. This strategy could be effective but increase adverse reactions in many ways. Furthermore, therapeutic approaches including retinal neuroprotective aspects or early time point disease management have not been well handled through either approach (anti-VEGF therapy or tyrosine kinase inhibitors). DR and AMD are complex metabolic diseases associated with various pathophysiological progressions depending on the disease stage. Therefore, the development and progression of diseases in turn cannot be easily prevented or protected by one therapeutic molecular target such as VEGF inhibition. In this regard, our current summary will enable a more comprehensive understanding of therapeutic approaches in DR and AMD (Figure 1). HIFs could work as a strong metabolic regulator as well as a VEGF inhibitor under selective pathologic conditions in ocular diseases. PPARα could improve lipid metabolism and modulate its relevant inflammatory process under metabolic dysregulation detected during ocular disease progression. Antioxidants could actively scavenge continuously recurring ROS that can acutely damage retinal neuronal cells. Furthermore, cell therapy can directly replace damaged tissue and/or abnormal cells.

We hope further evidence will be accumulated in terms of cellular, molecular, and drug delivery-based therapeutic interventions for DR and AMD development and progression.

## Figures and Tables

**Table 1:** Summary of different strategies discussed in this review for treating DR and AMD.

Treatment	Advantages	Disadvantages; Limitations
Hypoxia-inducible factor (HIF) inhibition	Strong efficacy in inhibiting diverse inflammatory cytokines and angiogenic factors including VEGF; modulation of HIF-mediated apoptosis; selective targeting of pathologic HIF expression under hypoxic conditions	Side effects for systemic HIF inhibition; a lack of capability of prolonged drug release for the long-term effects
Peroxisome proliferator-activator receptor alpha (PPARα) activation	Improvements of lipid metabolism under systemic metabolic dysregulation; anti-inflammation and anti-vascular damage; potent neuroprotective effects via the FGF21/PPARα pathway	Uncertainty about the direct or indirect therapeutic effects; a lack of identification of the target cell type in the eye under the disease condition
Cell therapy	Ocular protection as well as regeneration/replacement; the potential for personalized medicine	limited availability and accessibility; unexpected complications; a lack of experimental evidence; ethical issue; high cost
Antioxidants (lutein, zeaxanthin, and vitamins)	Strong efficacy to enzymatically scavenge ROS; direct reduction of oxidative stress-mediated retinal cell death or dysfunction; anti-inflammation under oxidative stress conditions	Potential interference with important physiologic functions in cells; the suitable antioxidants that remain unknown, depending on the disease states

## Data Availability

Data sharing is not applicable as no new data is generated.

## Abbreviations

The following abbreviations are used in this manuscript:

AMD	Age-related macular degeneration
PDGFR	Platelet-derived growth factor receptor
GA	Geographic atrophy
MNV	Macular neovascularization
AREDS	Age-Related Eye Disease Study
DR	Diabetic retinopathy
VEGF	Vascular endothelial growth factor
HIF	Hypoxia-inducible factor
PPAR	Peroxisome proliferator-activator receptor
FGF21	Fibroblast growth factor 21
OIR	Oxygen-induced retinopathy
SPPARMα	Selective peroxisome proliferator-activated receptor alpha modulator
SGLT2	Sodium-glucose cotransporter 2
TGF	Transforming growth factor
AGE	Advanced glycation end product
PKC	Protein kinase C
MCP-1/CCL2	Monocyte chemoattractant protein-1
TNF	Tumor necrosis factor
ANG	Angiopoietin
STZ	Streptozotocin
IL	Interleukin
CNV	Choroidal neovascularization
ROS	Reactive oxygen species
